# Supplementary figures and images for: Identification of key genes and validation of key gene aquaporin 1 on Wilms’ tumor metastasis
Source: PeerJ. 2023 Oct 26;11:e16025. doi: 10.7717/peerj.16025 (PMC10613441; doi:10.7717/peerj.16025)

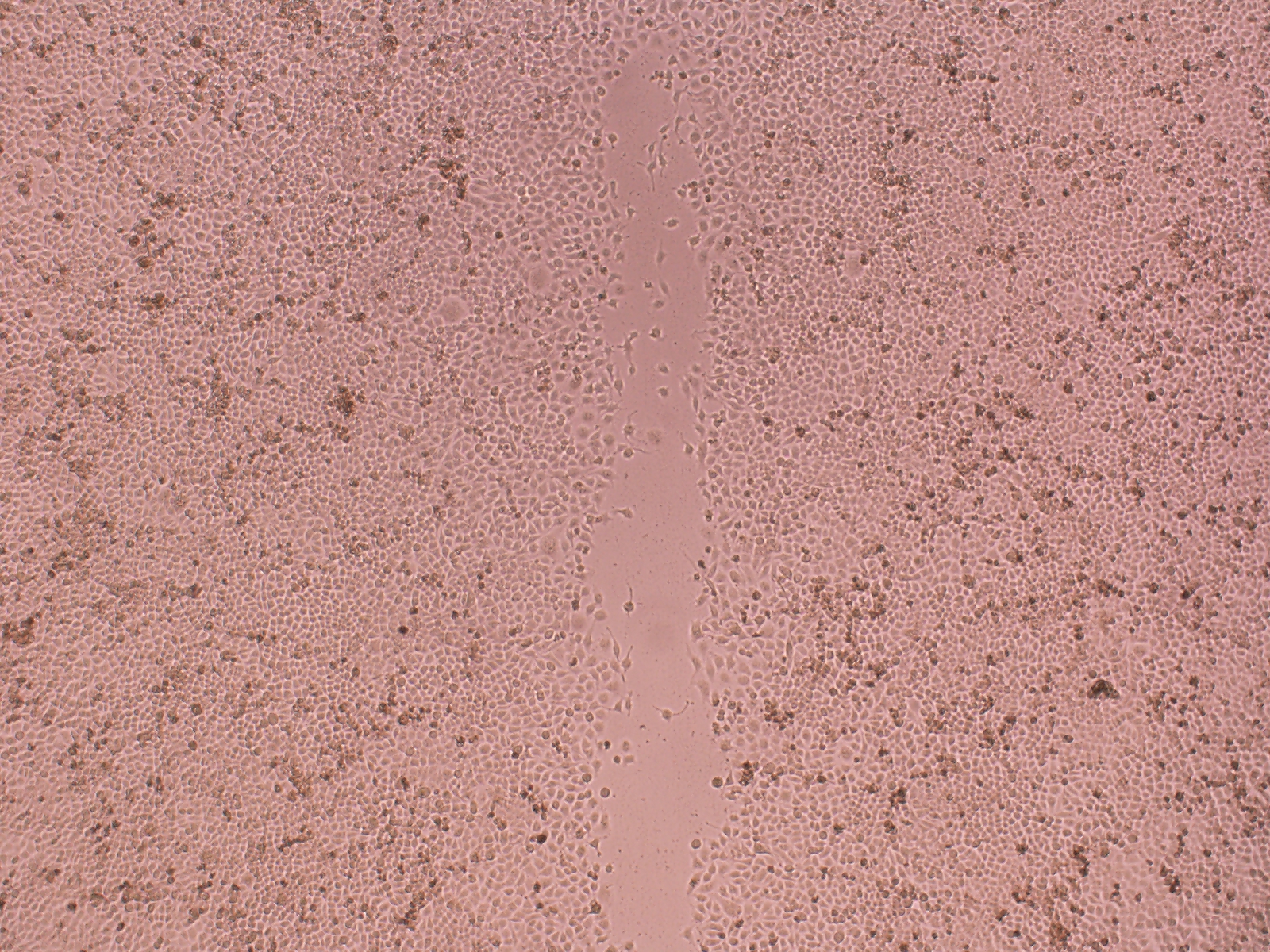

Supplement: Supplemental Information 10 [file peerj-11-16025-s010.zip › Wound Healing/negative control-24h.jpg]

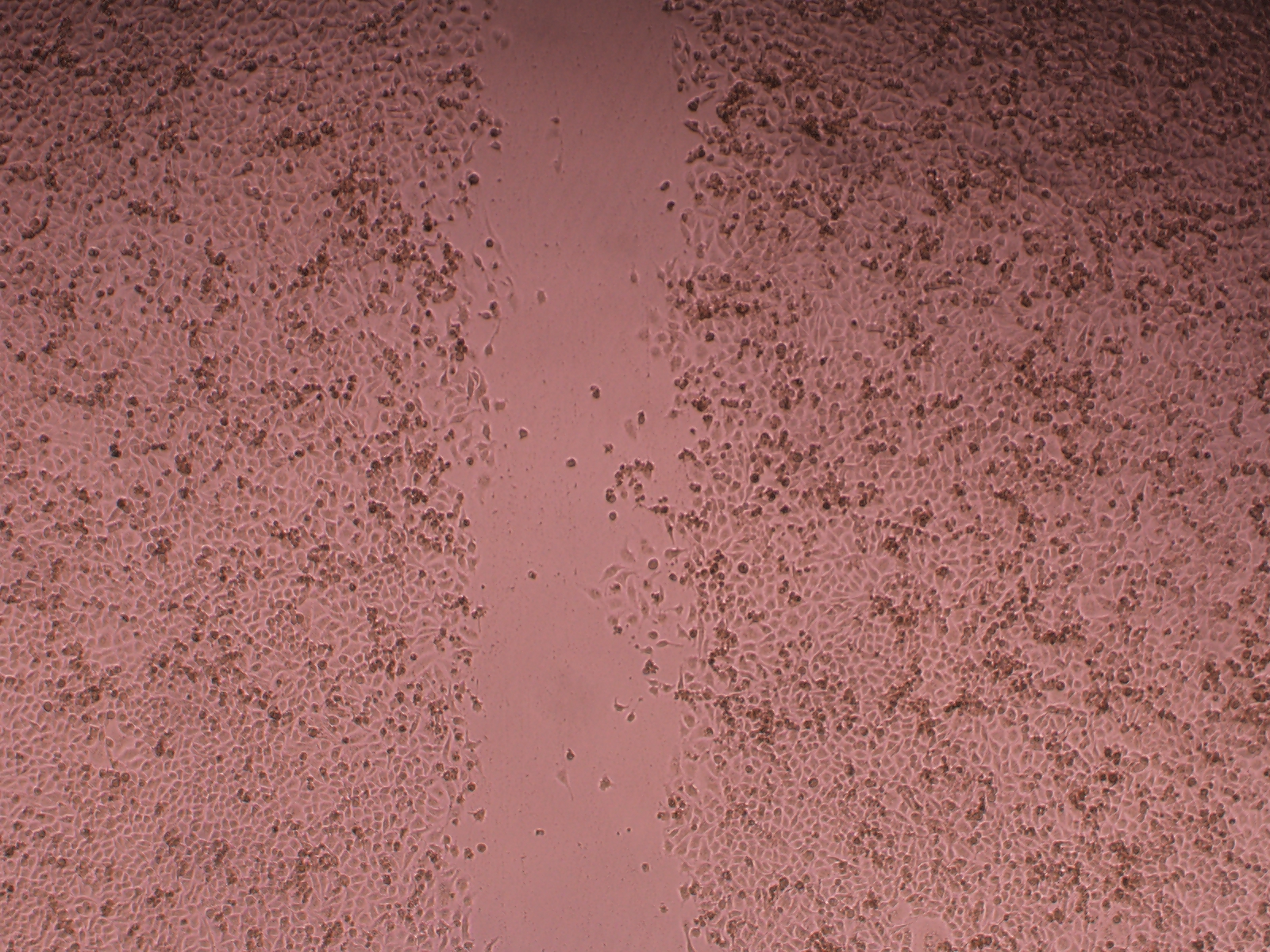

Supplement: Supplemental Information 10 [file peerj-11-16025-s010.zip › Wound Healing/over expression-24h.jpg]

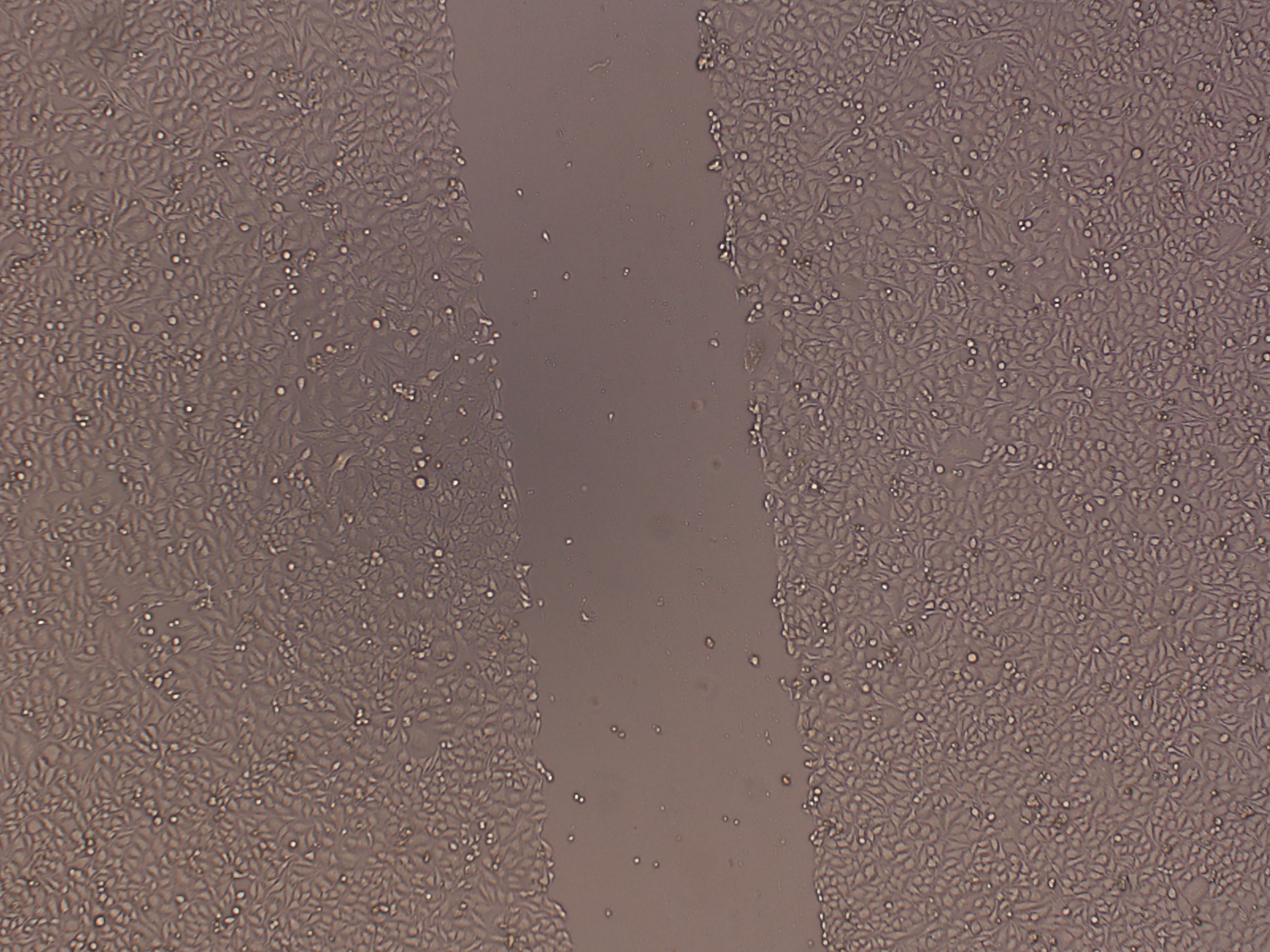

Supplement: Supplemental Information 10 [file peerj-11-16025-s010.zip › Wound Healing/negative control-0h.jpg]

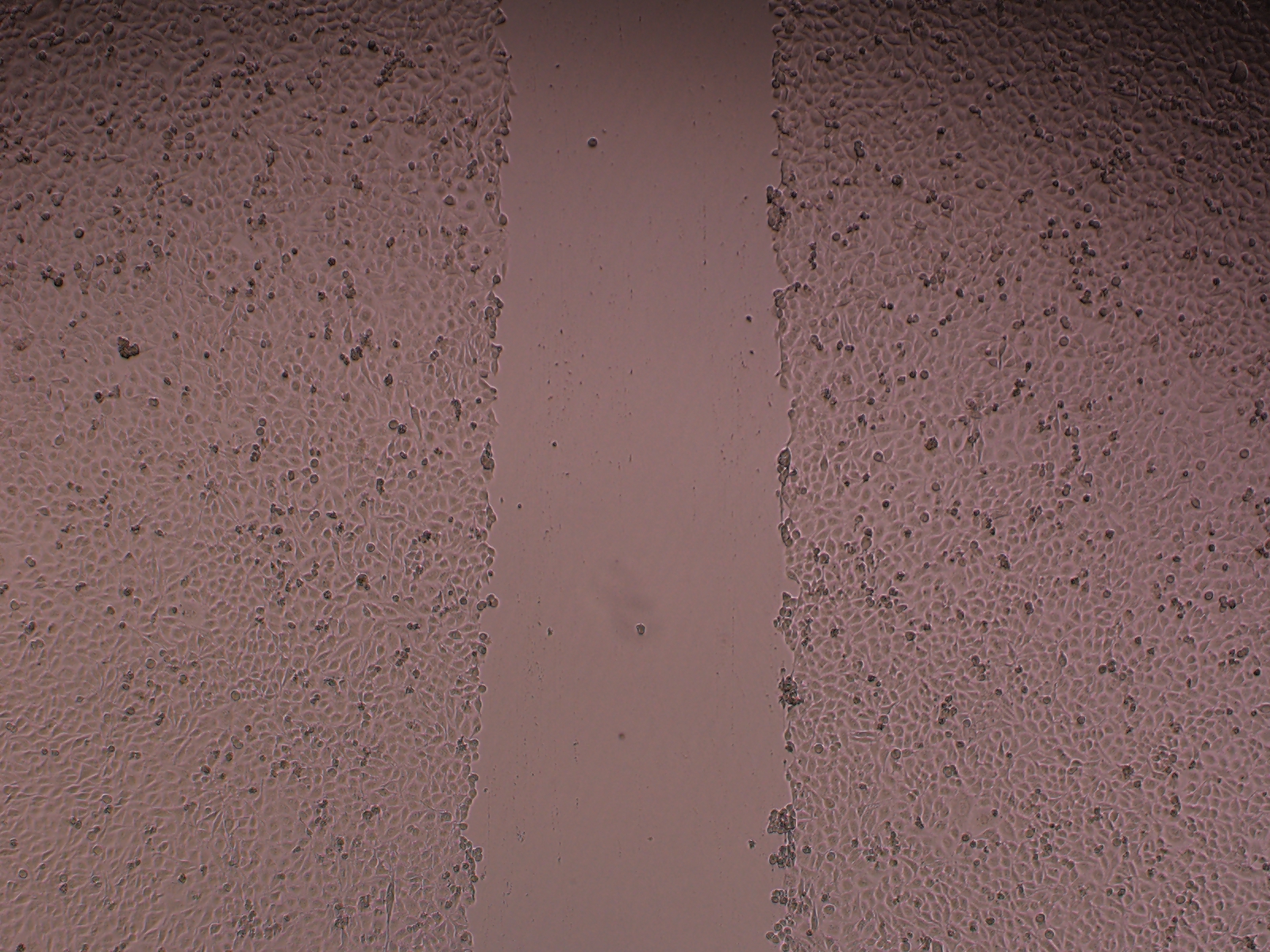

Supplement: Supplemental Information 10 [file peerj-11-16025-s010.zip › Wound Healing/over expression-0h.jpg]

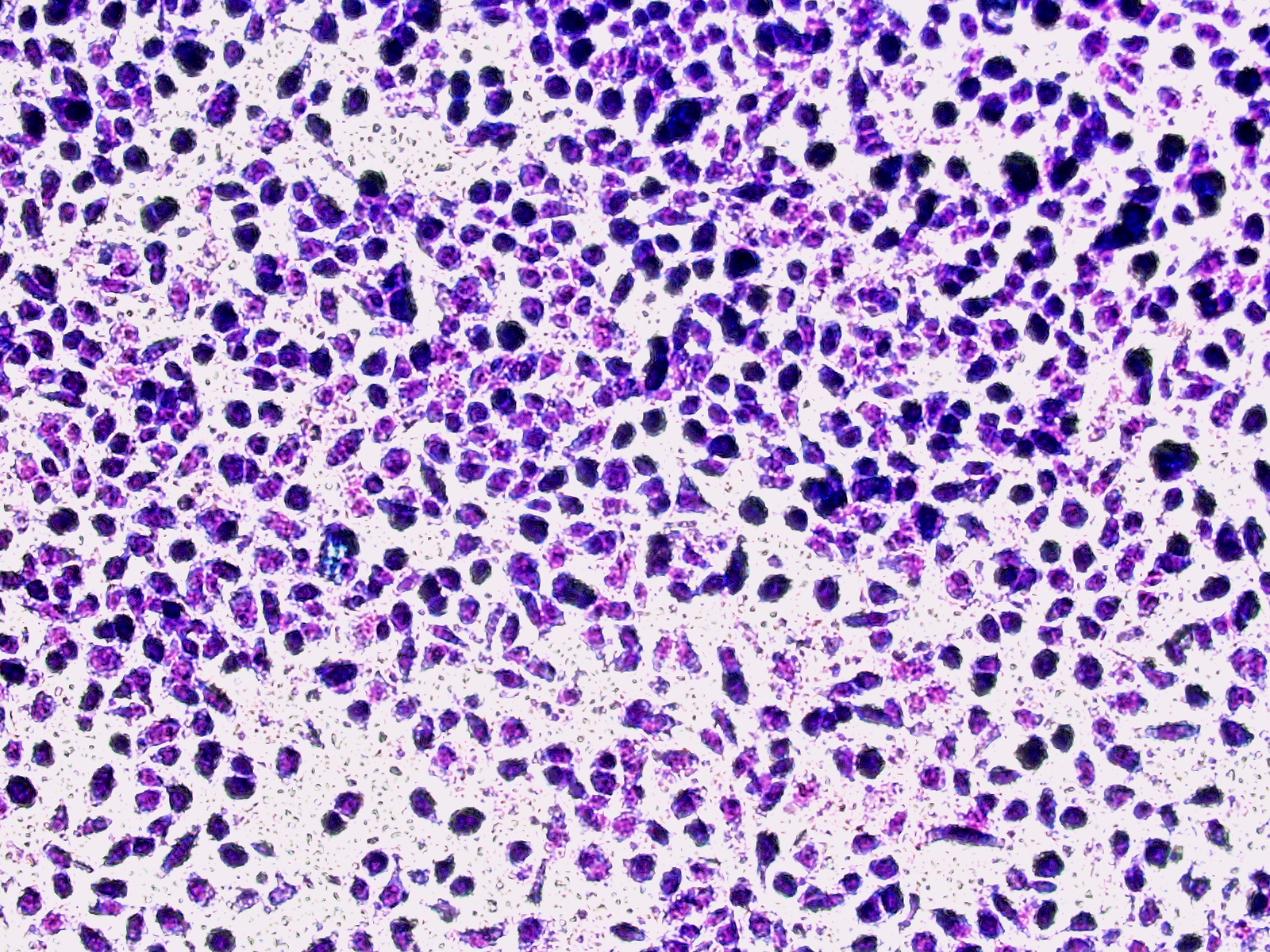

Supplement: Supplemental Information 11 [file peerj-11-16025-s011.zip › Invasion/negative control.tif]

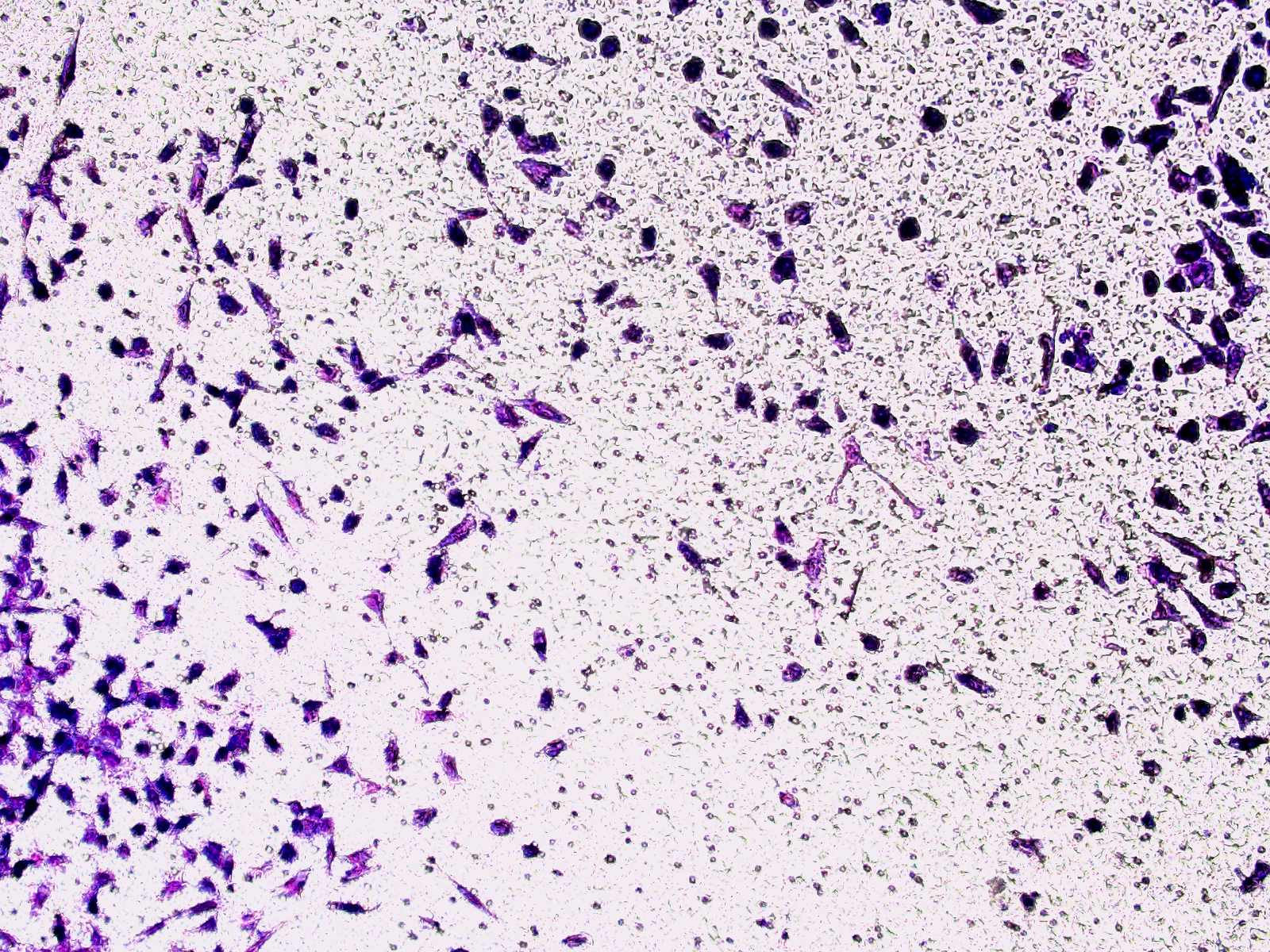

Supplement: Supplemental Information 11 [file peerj-11-16025-s011.zip › Invasion/over expression.tif]

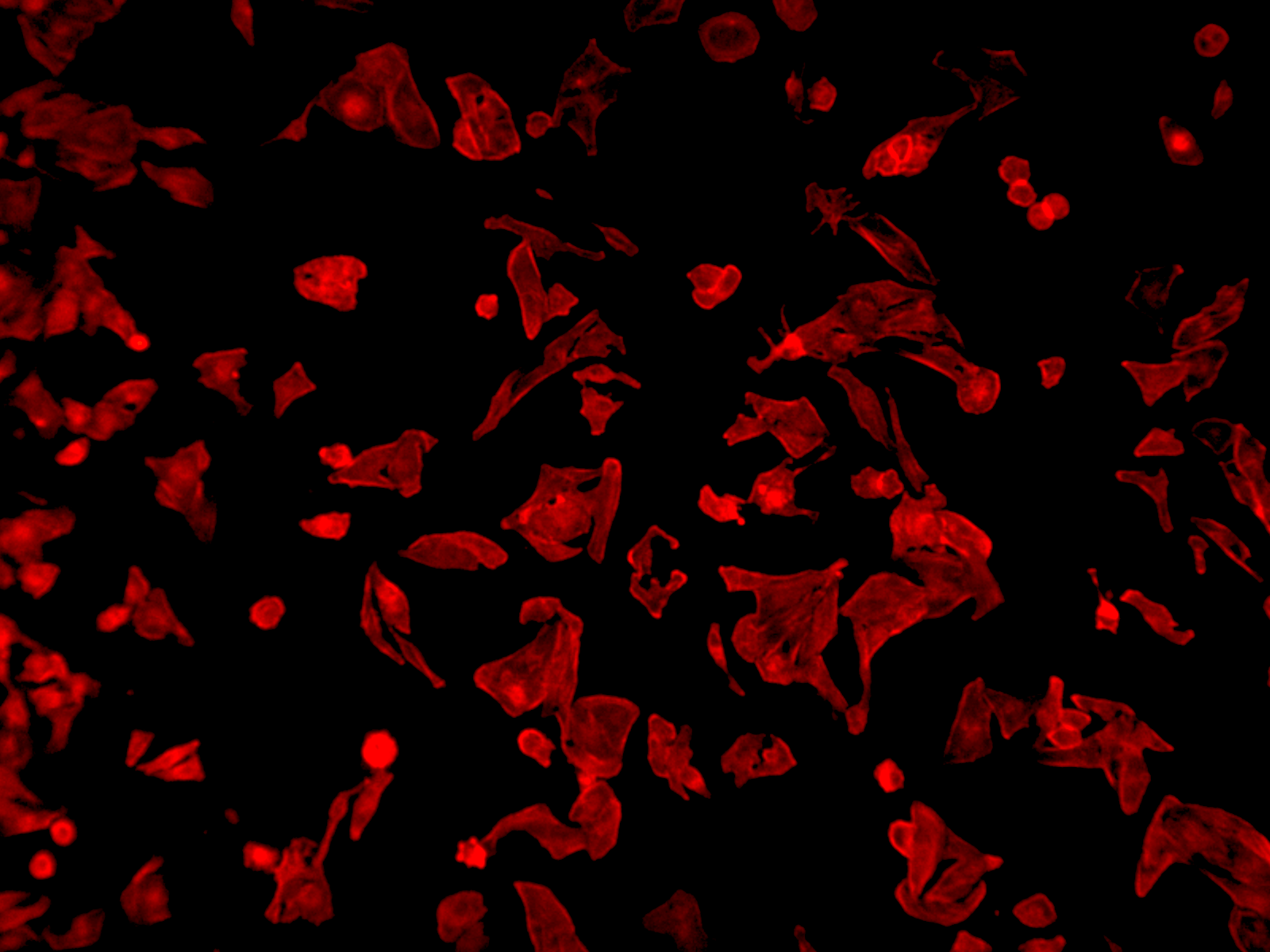

Supplement: Supplemental Information 12 [file peerj-11-16025-s012.zip › cytoskeleton/negative control.png]

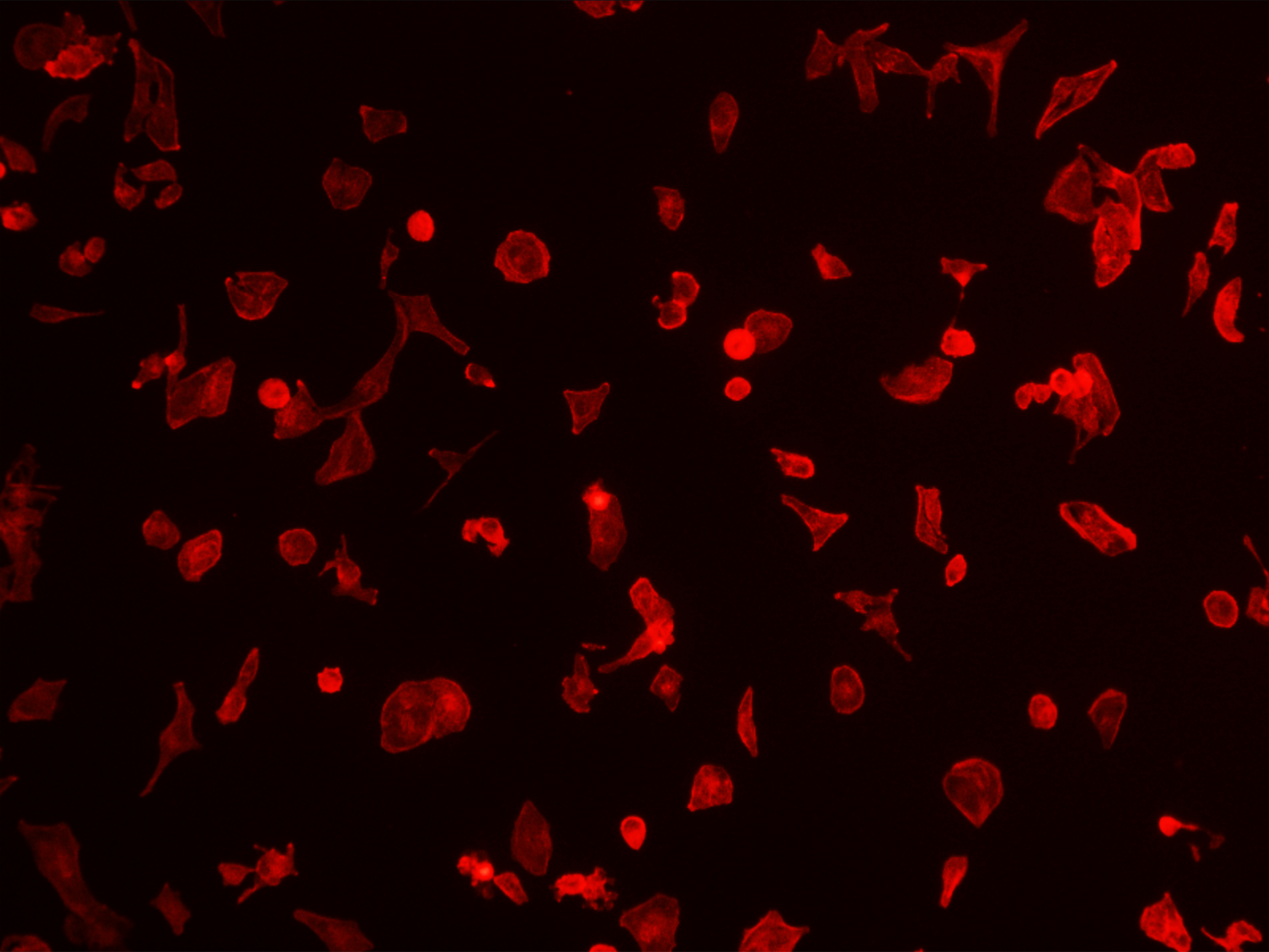

Supplement: Supplemental Information 12 [file peerj-11-16025-s012.zip › cytoskeleton/over expression.tif]

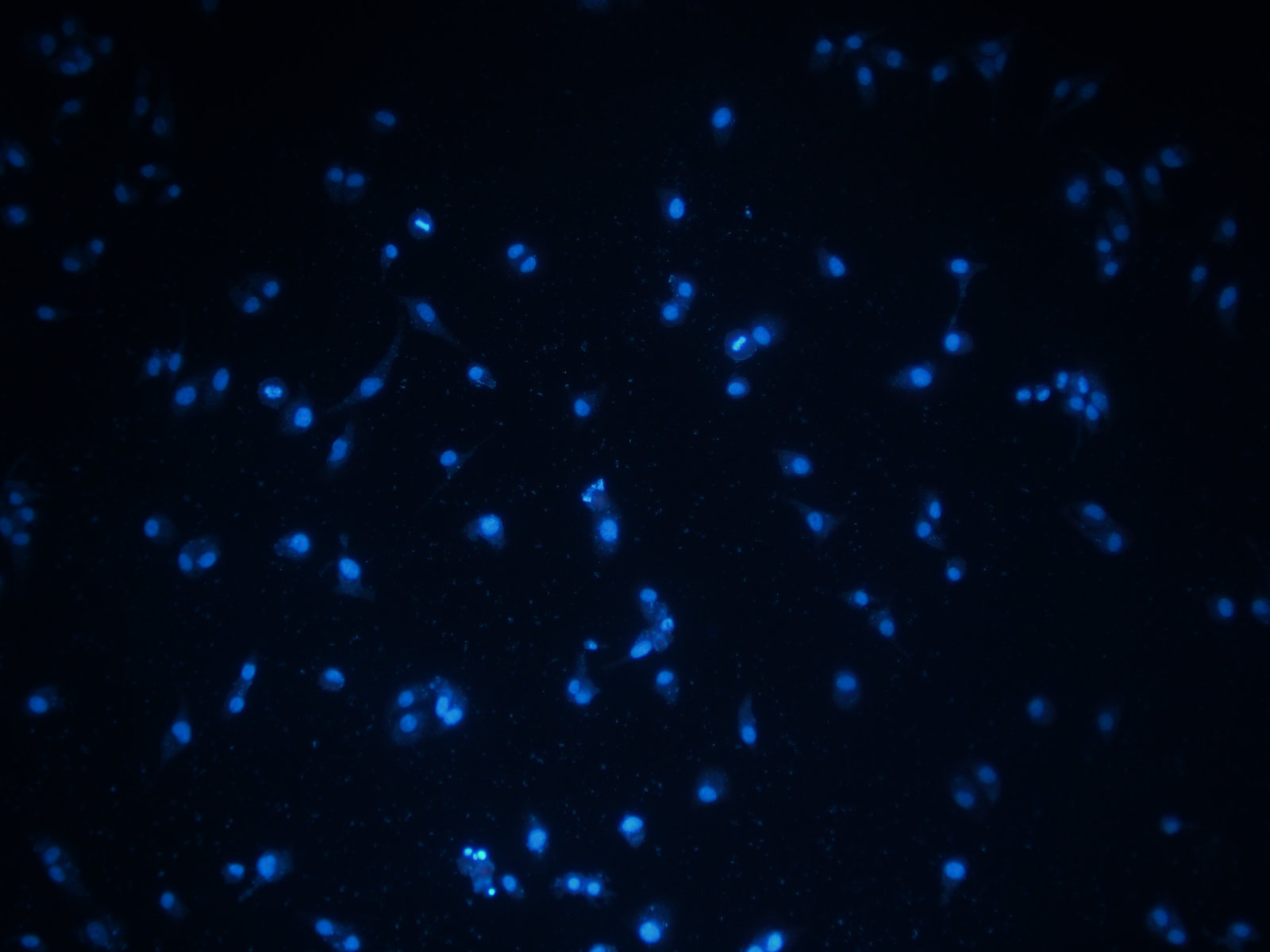

Supplement: Supplemental Information 12 [file peerj-11-16025-s012.zip › cytoskeleton/over expression-DAPI.png]

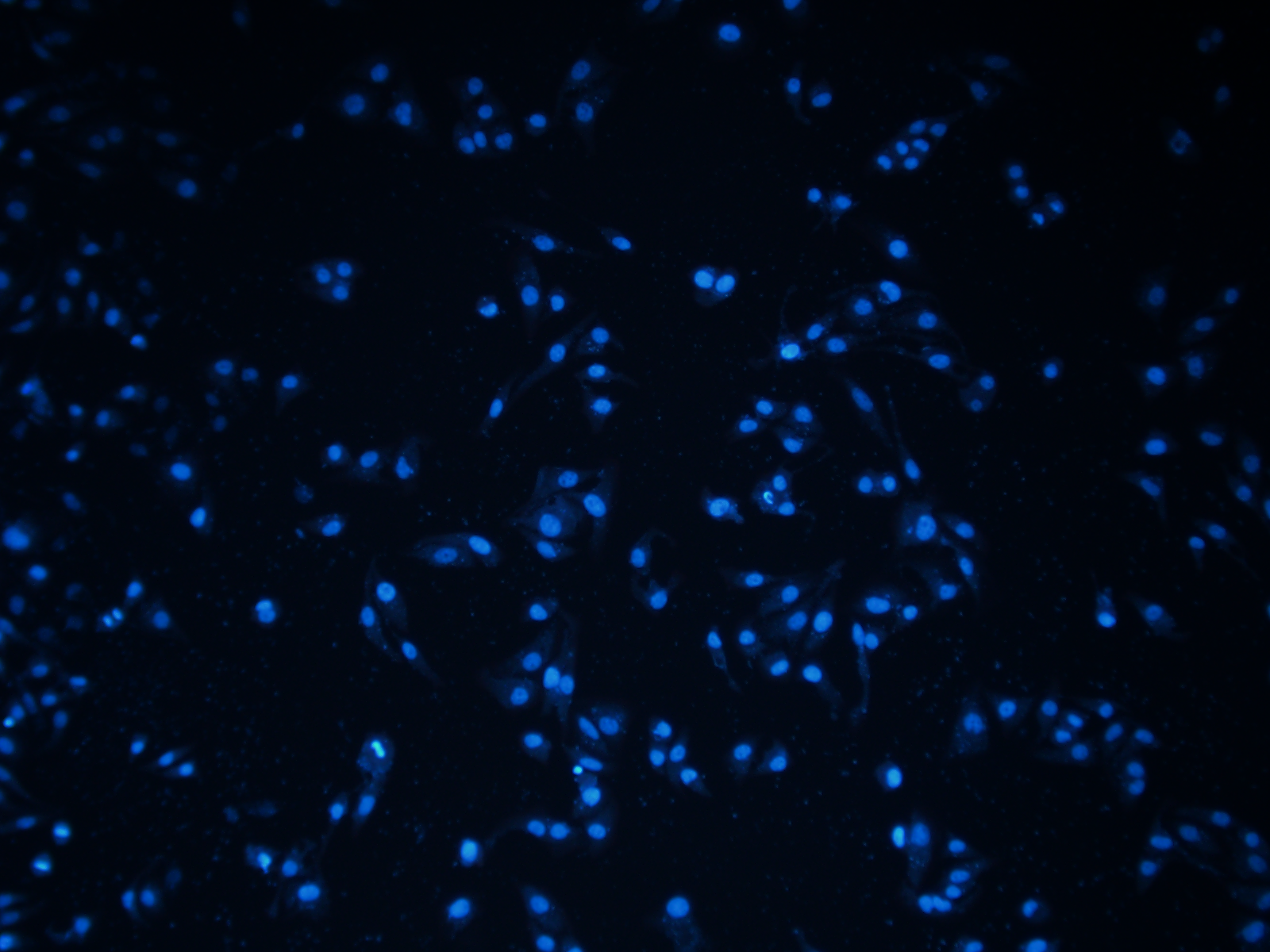

Supplement: Supplemental Information 12 [file peerj-11-16025-s012.zip › cytoskeleton/negative control-DAPI.png]

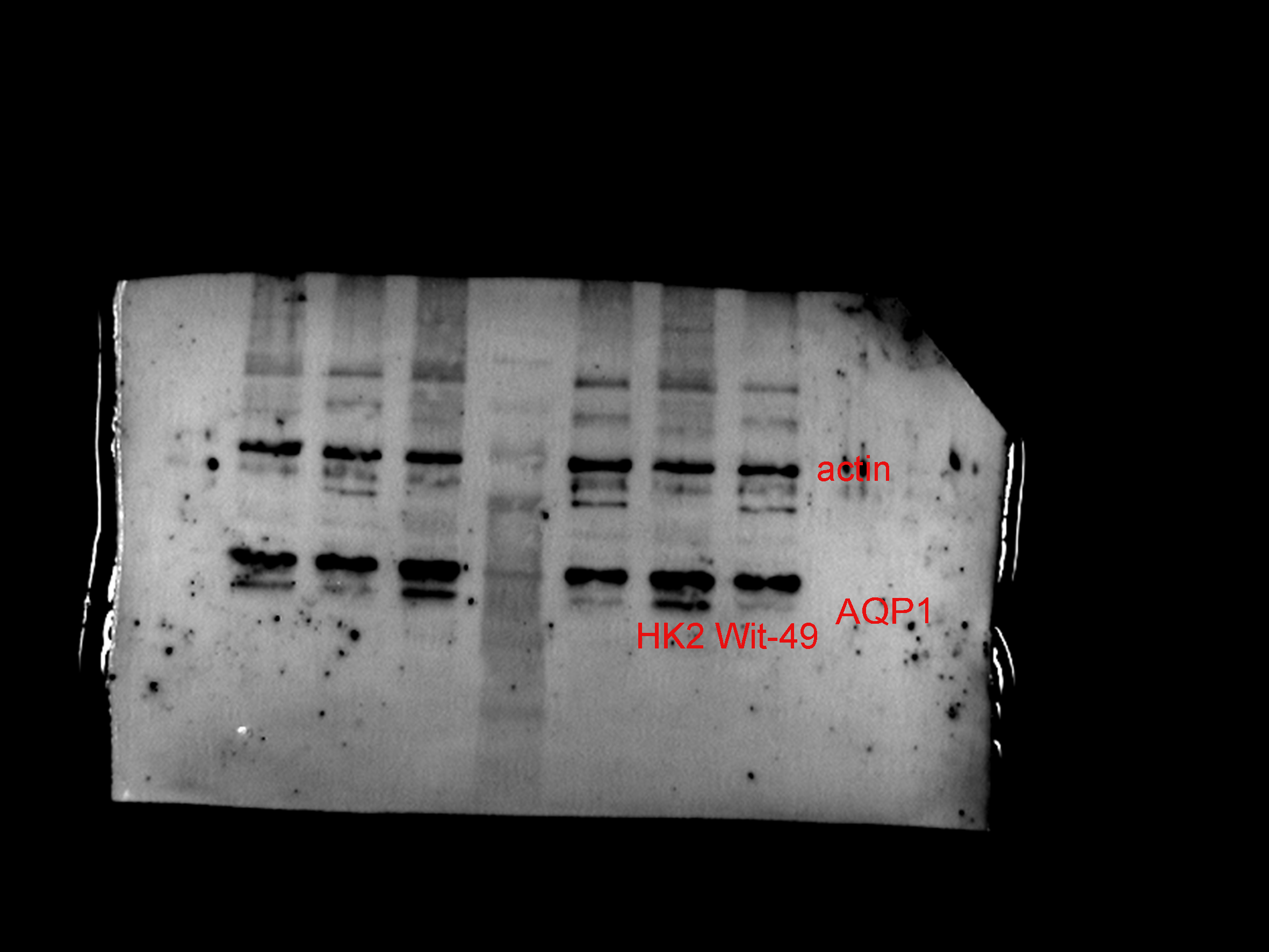

Supplement: Supplemental Information 13 [file peerj-11-16025-s013.zip › WB/AQP1-HK2-Wit-49.tif]

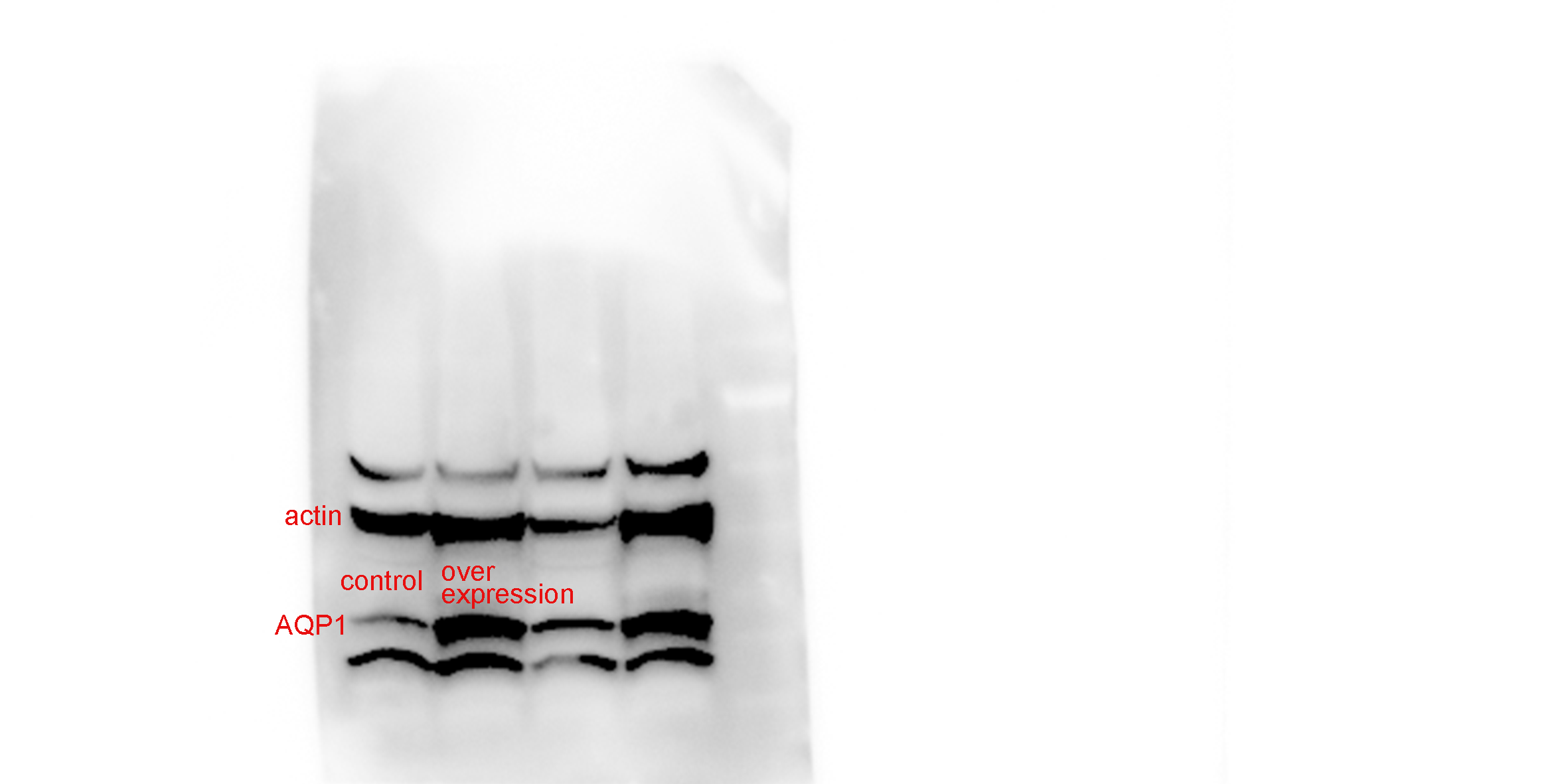

Supplement: Supplemental Information 13 [file peerj-11-16025-s013.zip › WB/aqp1+actin-negative control-over expression.tif]

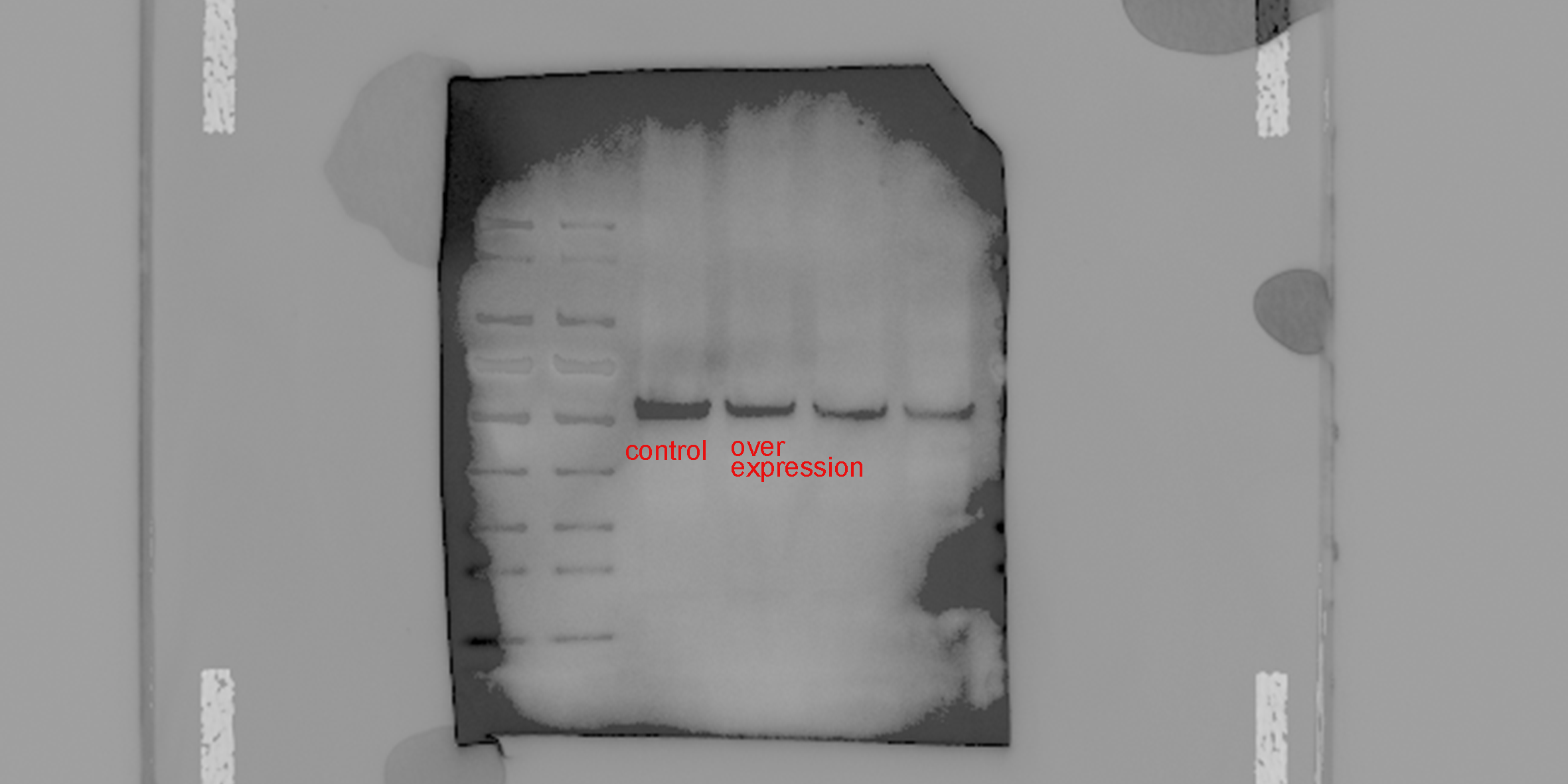

Supplement: Supplemental Information 13 [file peerj-11-16025-s013.zip › WB/Vemintin&MARKER.tif]

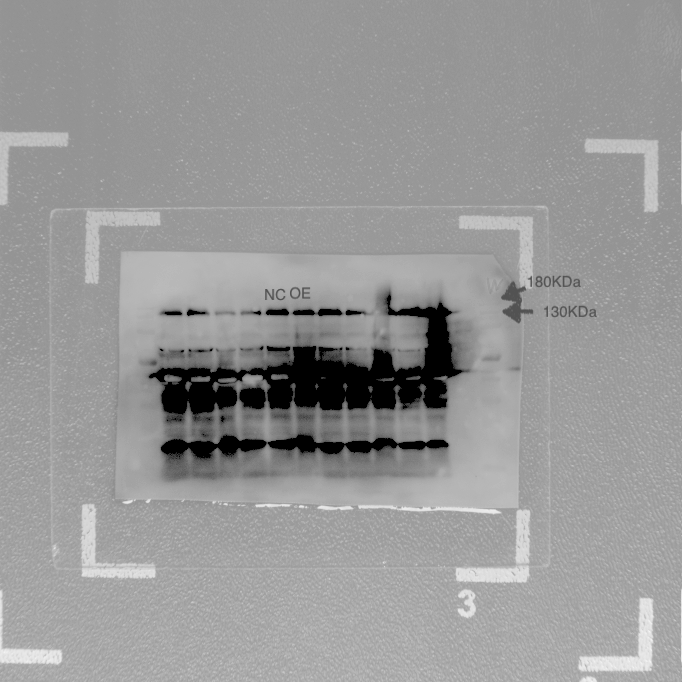

Supplement: Supplemental Information 13 [file peerj-11-16025-s013.zip › WB/W-N-candeherin.tif]

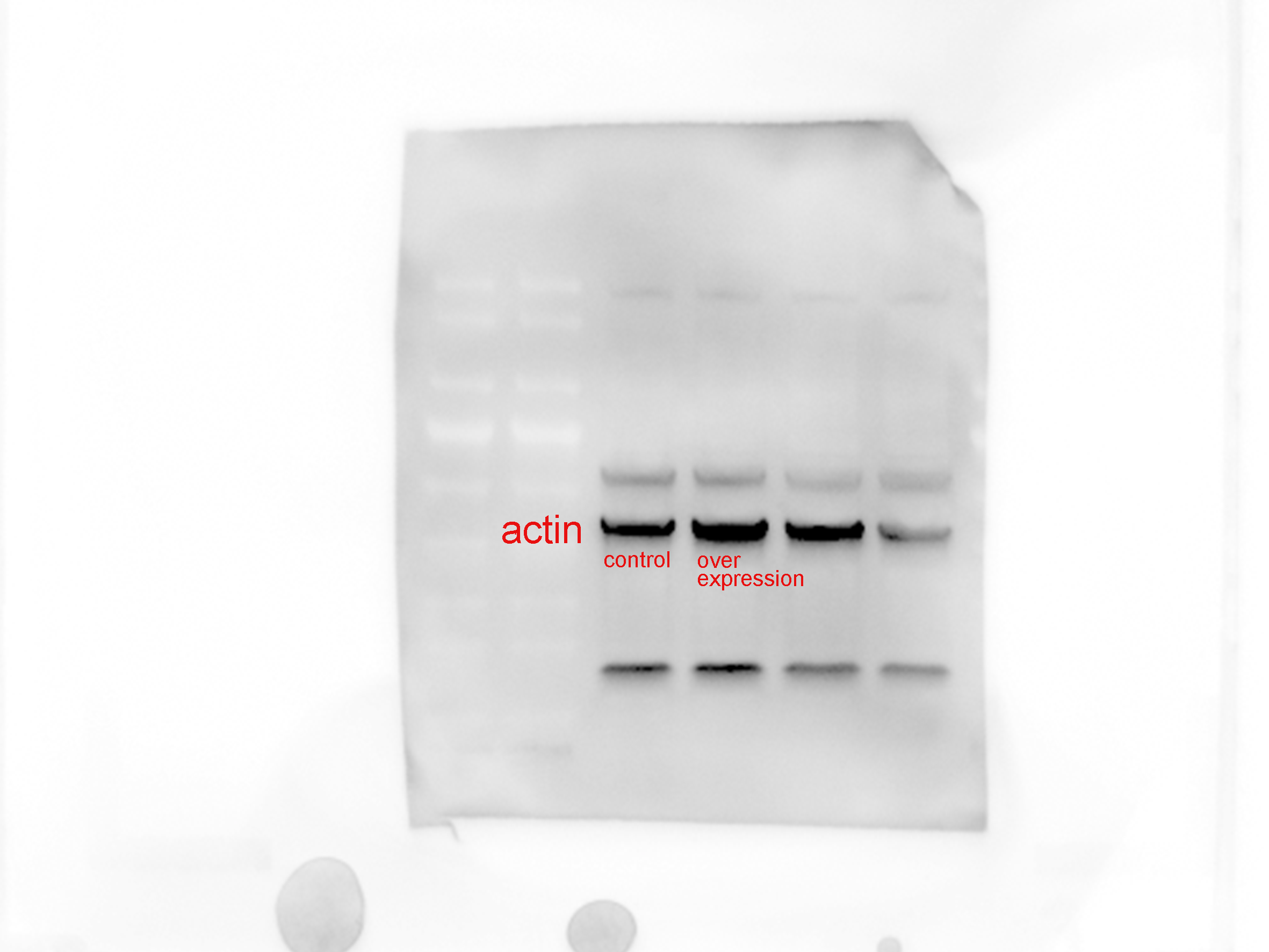

Supplement: Supplemental Information 13 [file peerj-11-16025-s013.zip › WB/actin-vemintin.tif]

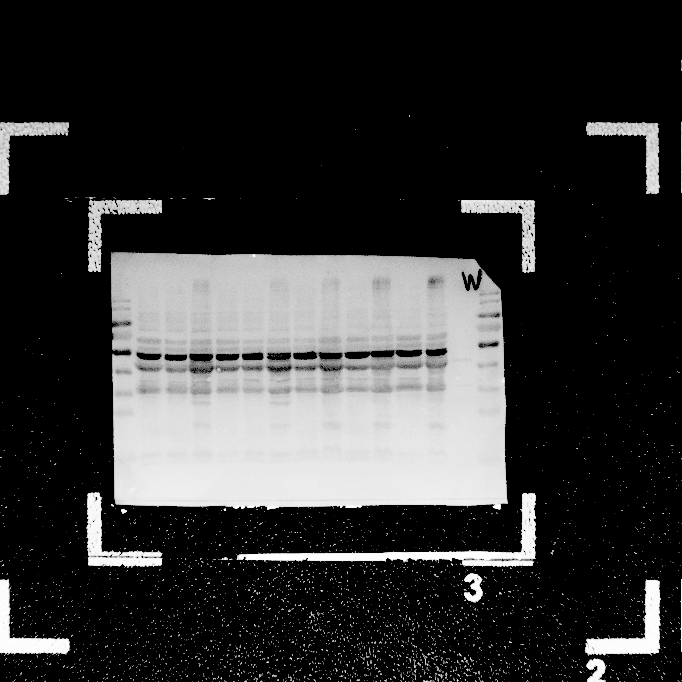

Supplement: Supplemental Information 13 [file peerj-11-16025-s013.zip › WB/E-Cadeherin-actin.tif]

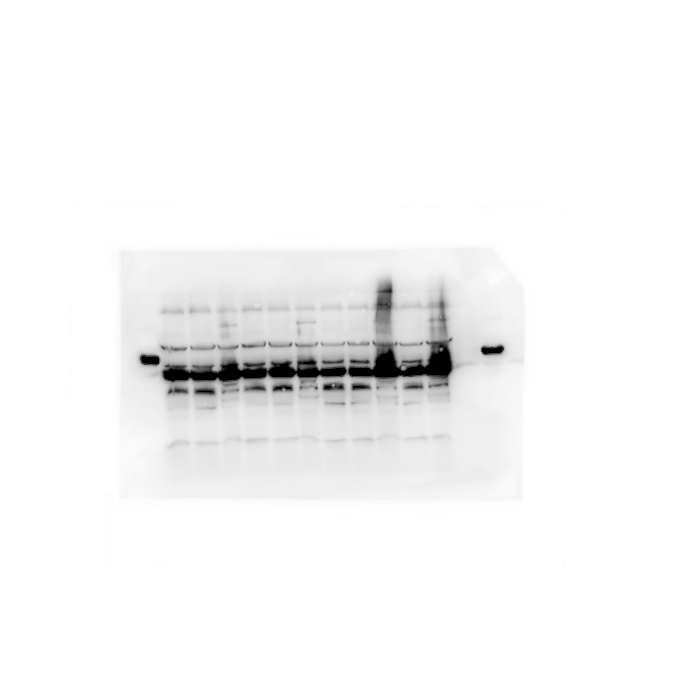

Supplement: Supplemental Information 13 [file peerj-11-16025-s013.zip › WB/wit-n-ca-actin.tif]

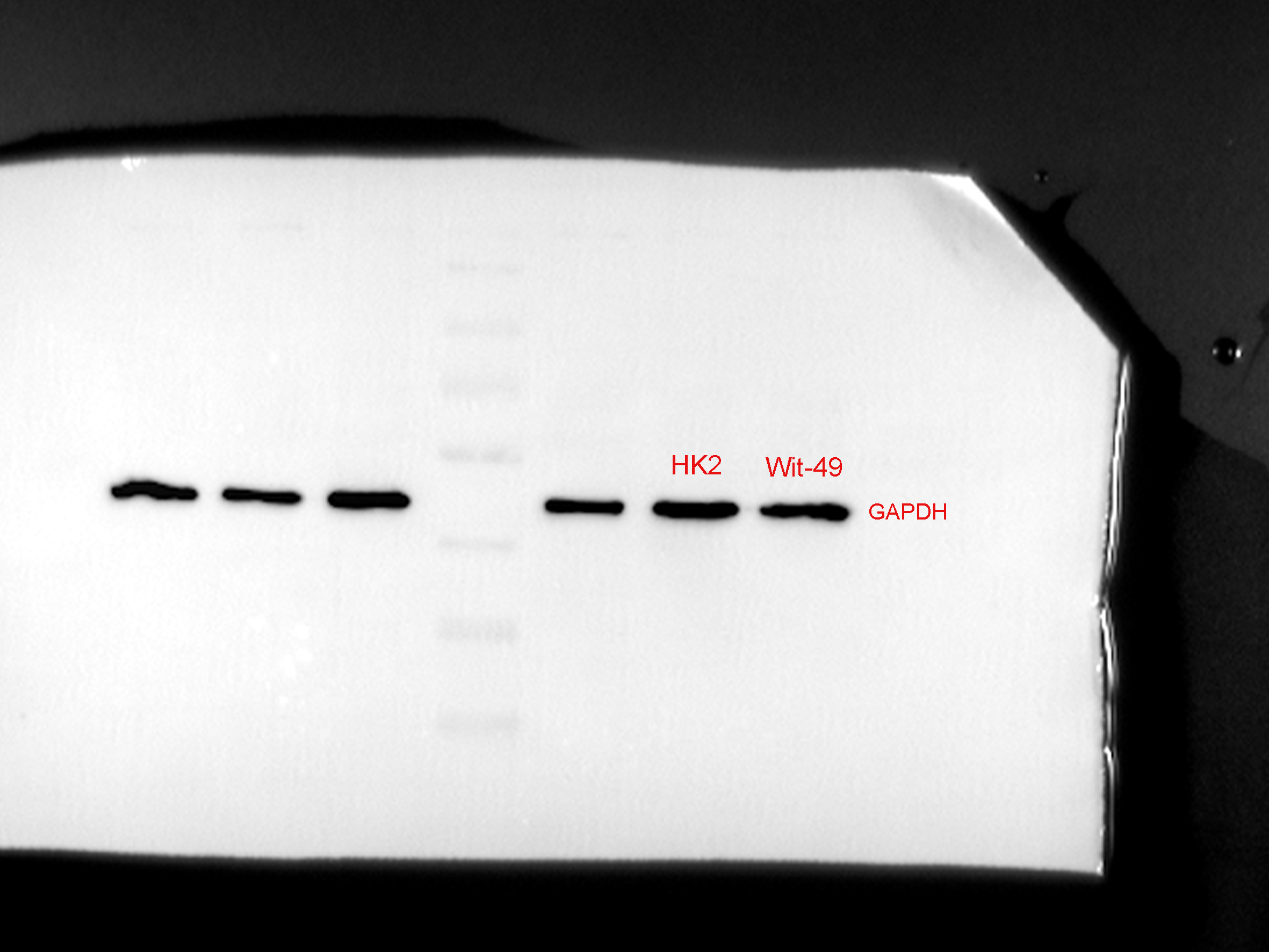

Supplement: Supplemental Information 13 [file peerj-11-16025-s013.zip › WB/GAPDH-AQP1-HK2&Wit-49.tif]

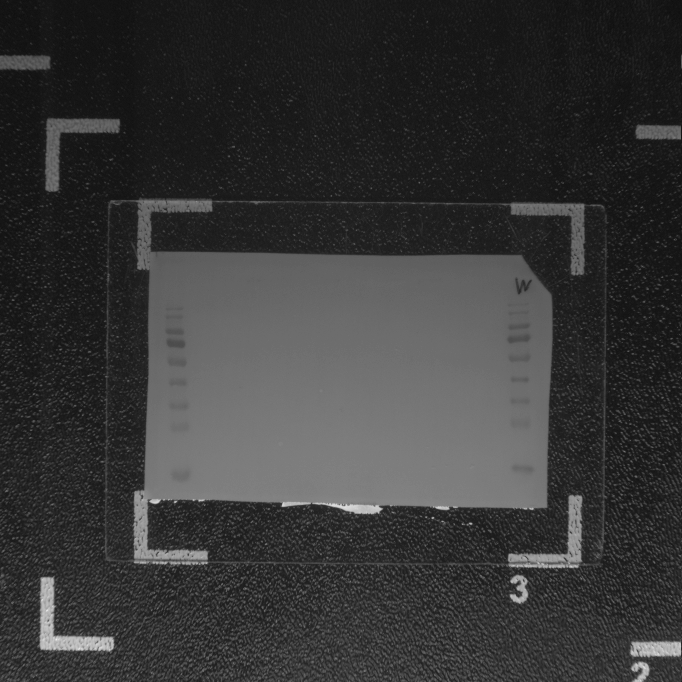

Supplement: Supplemental Information 13 [file peerj-11-16025-s013.zip › WB/WIT-N-CA-MARKER.tif]

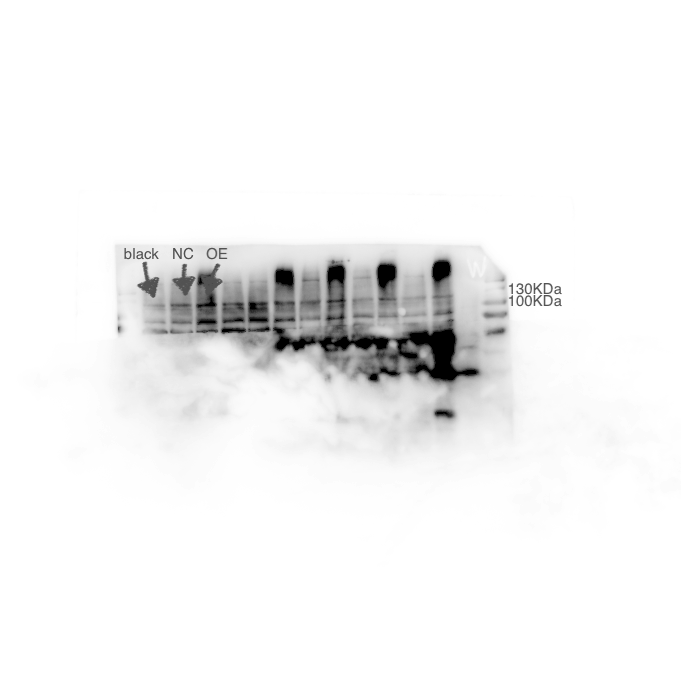

Supplement: Supplemental Information 13 [file peerj-11-16025-s013.zip › WB/wit-E-Cadeherin.tif]

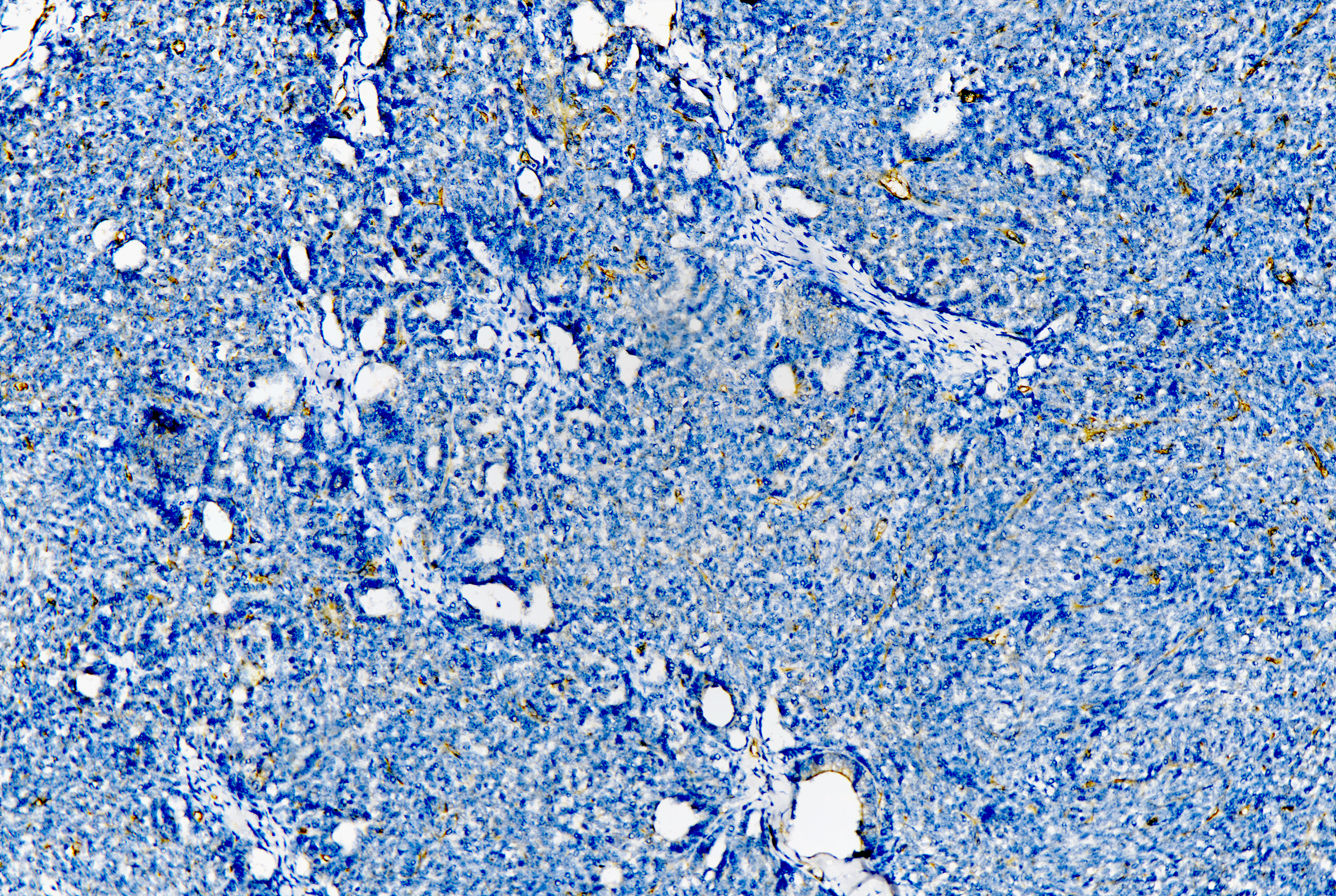

Supplement: Supplemental Information 14 — The magnification was 100. [file peerj-11-16025-s014.zip › IHC-100/WT 100σÇì.tif]

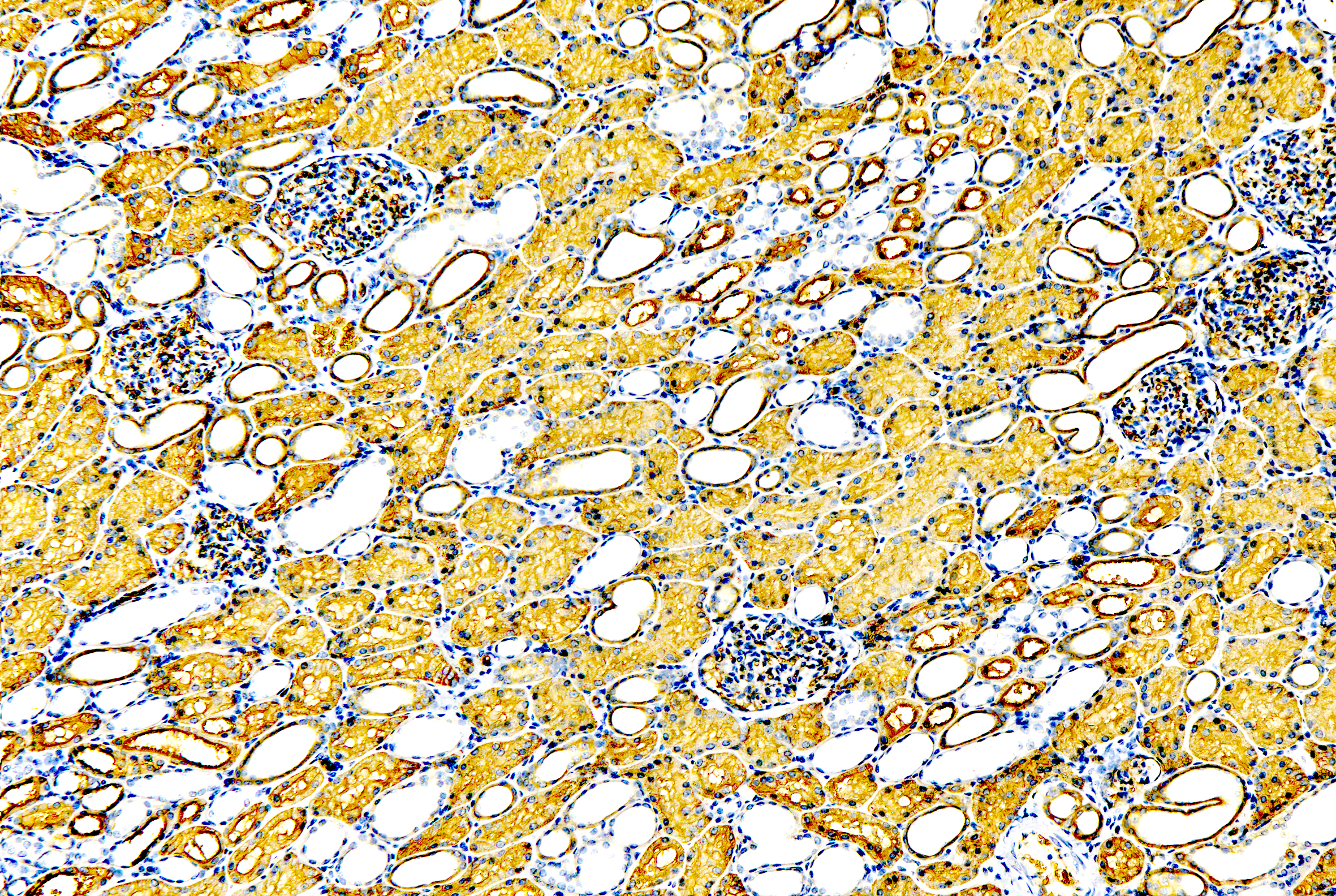

Supplement: Supplemental Information 14 — The magnification was 100. [file peerj-11-16025-s014.zip › IHC-100/normal 100σÇì.tif]

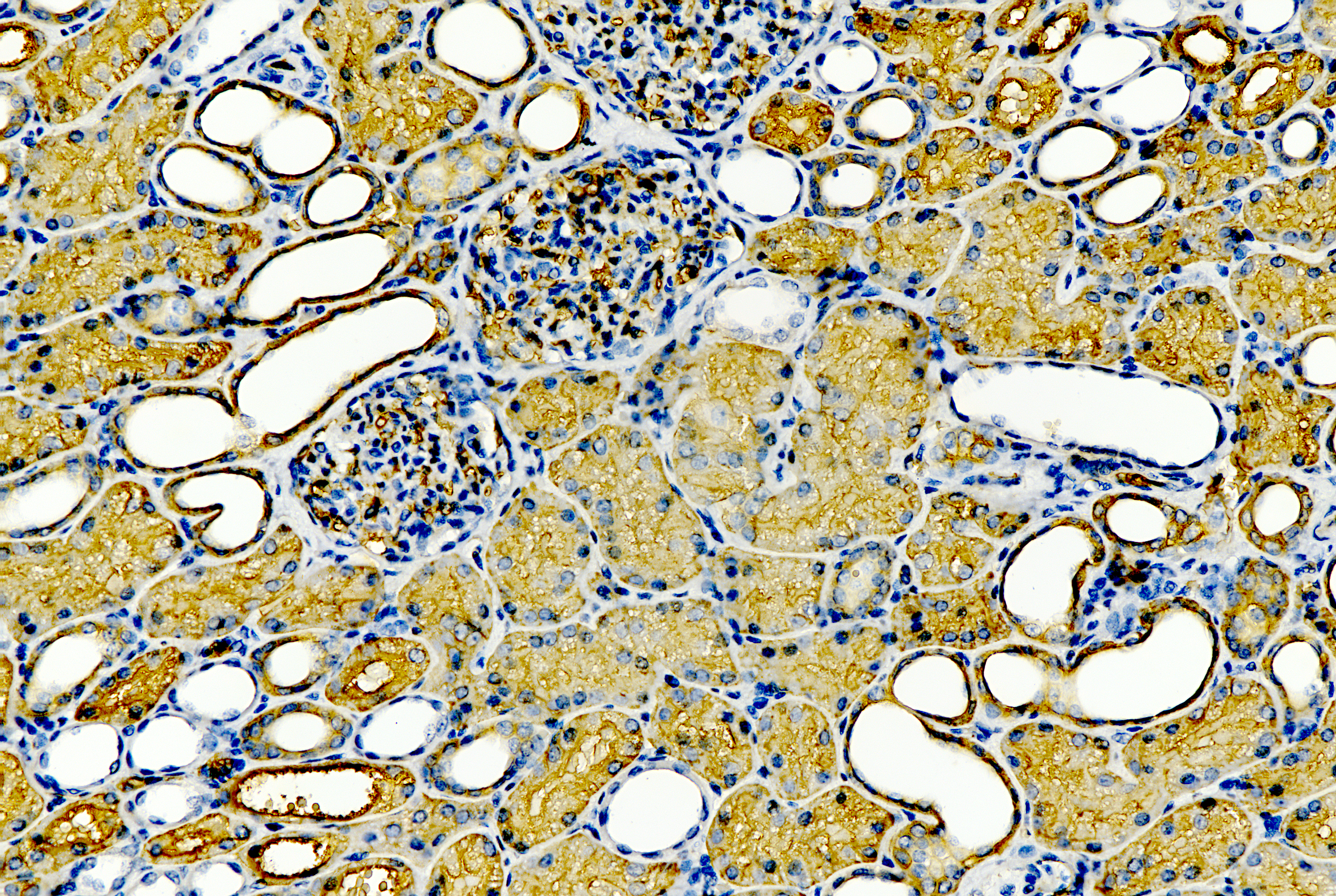

Supplement: Supplemental Information 15 — The magnification was 200. [file peerj-11-16025-s015.zip › IHC-200/normal 200σÇì.tif]

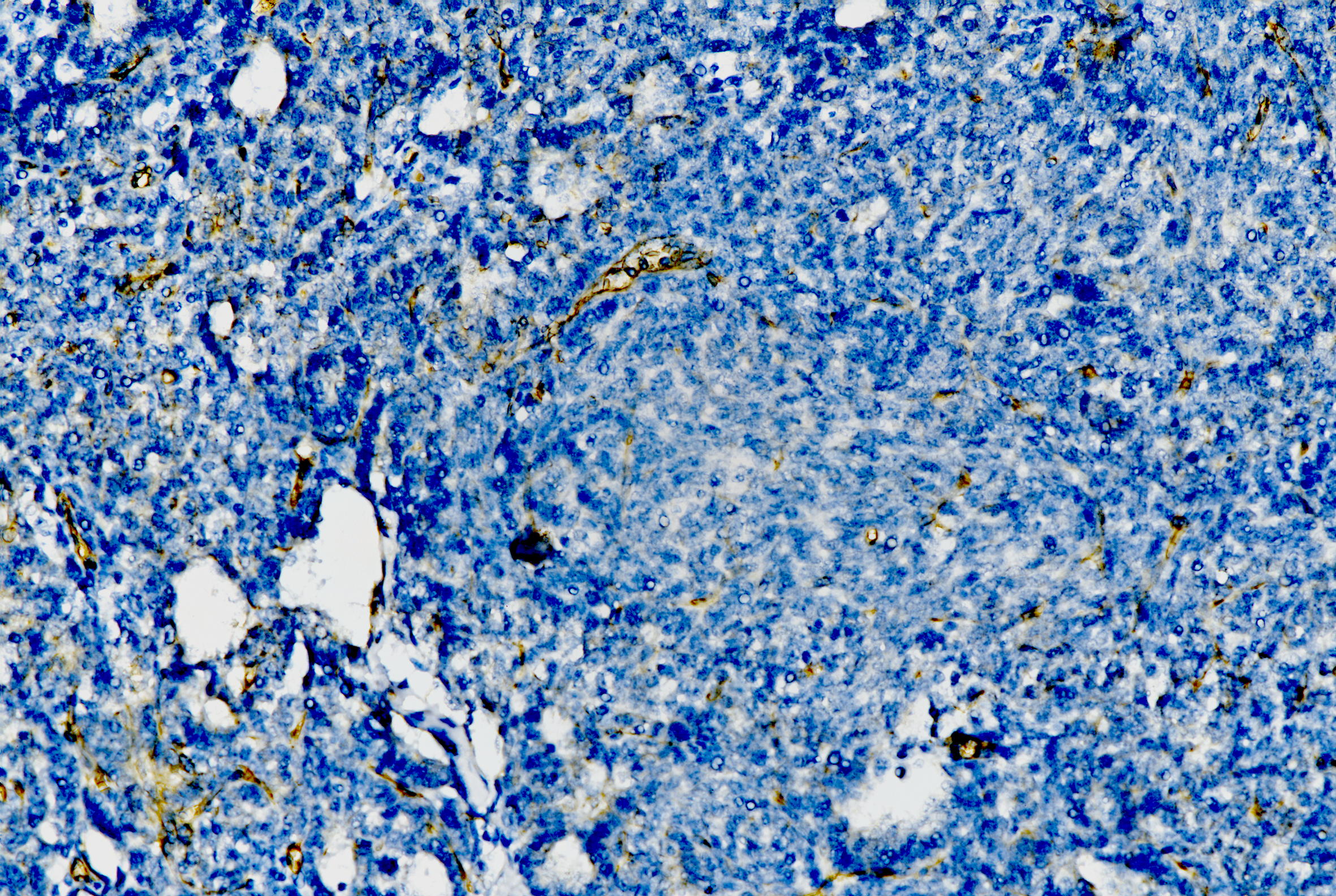

Supplement: Supplemental Information 15 — The magnification was 200. [file peerj-11-16025-s015.zip › IHC-200/WT 200σÇì.tif]
